# Inhibition of chemokine expression in rat inflamed paws by systemic use of the antihyperalgesic oxidized ATP

**DOI:** 10.1186/1471-2172-6-18

**Published:** 2005-07-22

**Authors:** Alessandro Fulgenzi, Giacomo Dell'Antonio, Chiara Foglieni, Elena Dal Cin, Paolo Ticozzi, Josè S Franzone, Maria Elena Ferrero

**Affiliations:** 1 Università degli Studi di Milano, Istituto di Patologia Generale, via Mangiagalli 31, 20133, Milano, Italy; 2 Ospedale S. Raffaele, via Olgettina 60, 20100, Milano, Italy; 3 Medestea Research and Production, via Magenta 43, 10128, Torino, Italy

## Abstract

**Background:**

We previously showed that local use of periodate oxidized ATP (oATP, a selective inhibitor of P2X7 receptors for ATP) in rat paw treated with Freund's adjuvant induced a significant reduction of hyperalgesia Herein we investigate the role of oATP, in the rat paws inflamed by carrageenan, which mimics acute inflammation in humans.

**Results:**

Local, oral or intravenous administration of a single dose of oATP significantly reduced thermal hyperalgesia in hind paws of rats for 24 hours, and such effect was greater than that induced by diclofenac or indomethacin. Following oATP treatment, the expression of the pro-inflammatory chemokines interferon-gamma-inducible protein-10 (IP-10), mon ocyte chemoattractant protein-1 (MCP-1) and interleukin-8 (IL-8) within the inflamed tissues markedly decreased on vessels and infiltrated cells. In parallel, the immunohistochemical findings showed an impairment, with respect to the untreated rats, in P2X7 expression, mainly on nerves and vessels close to the site of inflammation. Finally, oATP treatment significantly reduced the presence of infiltrating inflammatory macrophages in the paw tissue.

**Conclusion:**

Taken together these results clearly show that oATP reduces carrageenan-induced inflammation in rats.

## Background

We previously showed the ability of the local treatment with periodated oxidized ATP (oATP), an inhibitor of the P2X7 ATP (adenosine 5'-triphosphate) receptor [1], to relieve inflammatory pain in the rat paw, in which chronic inflammation was induced by Freund's complete adjuvant (CFA) injection [2,3]. There are two classes of ATP-receptors, the ionotropic P2X receptors and the G-protein-coupled P2Y receptors. Recently, these purinoceptors have been extensively studied because of their important roles in several ATP-mediated cellular functions [4]. In particular, there are seven subunits of P2X receptors (P2X_1–7_), which are differently expressed by many cell types [4]. Some P2X receptors are expressed in DRG neurons [5].

ATP, released by neuronal and non-neuronal cells, is able to initiate a pronociceptive signal through different P2X subtypes of P2 purinoceptors [6]. The expression of P2X by subsets of primary afferent neurons plays a role in the generation of pain from the periphery to the spinal cord [7,8]. ATP seems to be involved in the initiation of impulses in some sensory fibers [5]. In fact, excitation of sensory neurons by ATP evokes a sensation of pain in humans [9]. In rats, P2X receptor-mediated excitation of nociceptive afferents in normal and arthritic knee joints has been demonstrated [10]. Extracellular ATP exerts its activity during inflammatory processes. In fact, ATP is released by sensory nerves and by damaged cells during inflammation and acts by exciting primary sensory neurons [11,12].

P2X7 receptors for ATP have been demonstrated on monocytes/macrophages, dendritic cells, mast cells, fibroblasts and lymphocytes [13]. Such receptors mediate the ATP cytolytic activity on macrophages [14,15]. We showed the presence of P2X7 receptors also on subcutaneous sensory nerve terminals [3]. Thus, we hypothesized that local antihyperalgesic effect of oATP was related to the inhibition of pain transmission through the block of P2X7 receptors, due to oATP.

In the present work we investigated the effect of oATP treatment in the acutely carrageenan-inflamed rat hind paws. The treatment was performed locally, orally, or intravenously. The expression of the pro-inflammatory chemokines interferon-gamma-inducible protein-10 (IP-10), monocyte chemoattractant protein-1 (MCP-1) and interleukin-8 (IL-8) in the paws was assessed. IP-10 is a CXC-chemokine which regulates the recruitment of T cells [16,17]. It has inhibitory activity on fibroblast migration [18] and has been recently demonstrated to be a potent angiostatic protein in vivo [19]. MCP-1 is a CC-chemokine involved in the recruitment and activation of monocyte and macrophage lineage cell; it is high in serum of patients affected by both relapsing polychondritis and rheumatoid arthritis [20]. It has been also recognized as an angiogenic chemokine through the induction of VEGF (vascular endothelial growth factor) -A gene expression [21]. IL-8 is a CXC-chemokine which exerts potent chemotacting and stimulating activities on neutrophils. In fact, it causes the neutrophil degranulation and the generation of reactive oxygen intermediates, which are able to cause tissue damage and to amplify the inflammatory response [22]. We choosed to examine the expression of IL-8 because it is modulated during acute, carrageenan-induced inflammation, characterized by neutrophil recruitment. However, we also examined the expression of IP-10 and MCP-1, which recruit T cells and monocytes/macrophages, respectively, because such chemokines are released by mastocytes, macrophages and fibroblasts resident in the inflamed tissue. Moreover, some inflammatory mediators (histamine, thrombin, and cytokines as TNFα) produced during acute inflammation can activate endothelial cells, thus enabling them to produce and release MCP-1 [23]. In parallel with chemokine expression, we investigated the expression of P2X7 receptors and the presence of macrophage infiltrate in the paw tissue. Finally, we evaluated the antinociceptive effect of oATP in comparison with that obtained by two known antiinflammatory drugs, e.g. diclofenac and indomethacin. Our results clearly show that oATP exerts both antinflammatory and antihyperalgesic effects.

## Results

### Therapy with oATP reduces thermal hyperalgesia in rats

To test the potential role of oATP in reducing thermosensation *in vivo*, we subjected rats to the paw withdrawal latencies assay, and measured the intervals expressed in second. In untreated rats the values of paw withdrawal latencies averaged 12.0 ± 2.0, and were completely similar to those obtained in paws locally treated with saline (11.9 ± 1.0). oATP, administered using three different routes, did not significantly influence such data (12.0 ± 0.8, when administered locally; 11.9 ± 0.7, orally; 12.2 ± 0.9, intravenously; n = 7).

Following oATP administration, the rats were fully awake and responsive to stimuli.

**Figure 1:**
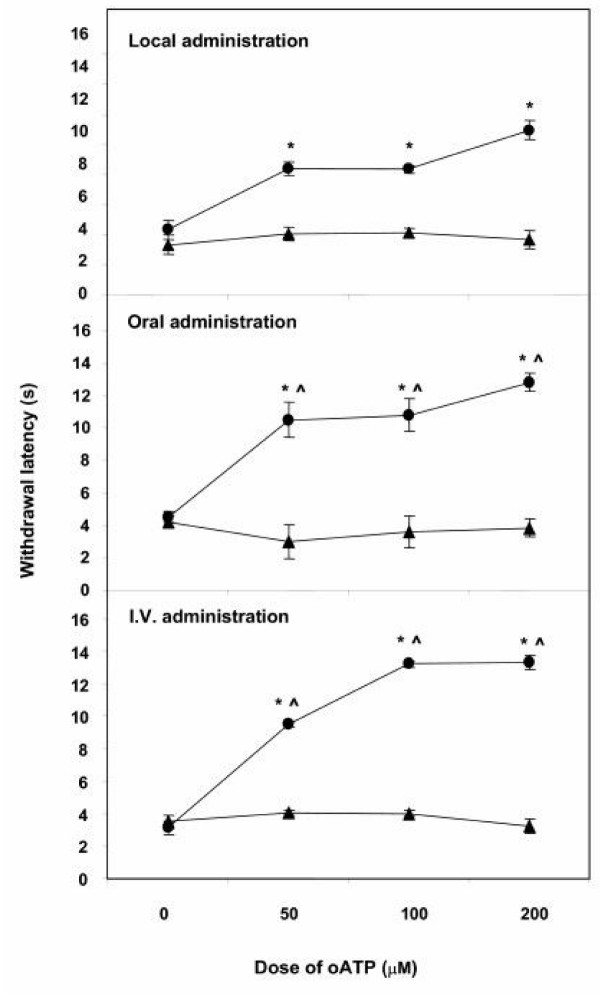
Reduction by oATP (used at the doses of 50, 100 and 200 μM, dissolved in 0.15 ml saline) of inflammatory pain in rat paws, as measured by paw withdrawal latency to a radiant heat stimulus, following the administration of carrageenan. Results are expressed as the mean ± SEM with n = 7 rats per group. * p < 0.05 compared with dose 0; ^ p < 0.05 compared with local administration at the same dose. All values were significantly higher in oATP-treated paws (circle) than in saline-treated paws (triangle).

**Figure 2:**
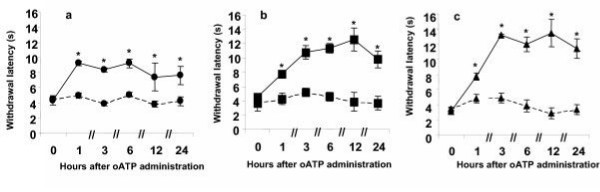
a)Withdrawal latencies measured 3 hours after intraplantar carrageenan injection (basal measurement = time 0) and at different hours following oATP (100 ( μM in 0.15 ml saline, continuous line) or 0.9% saline (0.15 ml, hatched line) local intraplantar injection (circle). Mean ± SEM of 7 rats. * p < 0.05 compared to saline at the same time. b) Withdrawal latencies measured 3 hours after intraplantar carrageenan injection (basal measurement = time 0) and at different hours following oATP (100 μM in 0.15 ml saline, continuous line) or saline (0.15 ml, hatched line) oral administration (square). Mean ± SEM of 7 rats. * p < 0.05 compared to saline at the same time. c) Withdrawal latencies measured as previously (3 hours after local carrageenan injection = time 0) and at different hours of oATP (100 μM in 0.15 ml saline, continuous line) or saline (0.15 ml, hatched line) intravenous administration (triangle). Mean ± SEM of 7 rats. * p < 0.05 compared to saline at the same time.

oATP administration reduced hind paw thermal hyperalgesia. Different doses of oATP dissolved in 0.15 ml saline (50, 100, 200 μM) were administered either intraplantarly, orally or intravenously 3 hours after carragenaan injection into right hind paws. Paw withdrawal latencies were measured 3 hours after oATP or saline (vehicle) administration. oATP treatments all significantly increased the antinociceptive score as revealed by an increase in the withdrawal latency compared to basal measurement (= 0 dose) (Fig. 1). Local oATP treatment was significantly less efficient than both oral and intravenous treatments in reducing hyperalgesia (Fig. 1). We decided to use the minimal dose displaying the maximal intravenous effect (e.g. 100 μM) of oATP in the following experiments. Time 0 corresponds to oATP injection. Local intraplantar injection of oATP in rat inflamed paws (3 hours after carrageenan injection) induced a significant increase in paw withdrawal latencies after 1 hour of treatment. Such increase was maintained in time (e.g. at 3, 6, 12 and 24 hours from treatment) compared with time 0 (Fig. 2a). Oral and intravenous administration of oATP induced significantly higher antihyperalgesic effect than local injection. Such effect was very evident after 3 hours of treatment, improved in the following 9 hours and slightly decreased after 24 hours (Fig. 2b and 2c, respectively)

**Figure 3:**
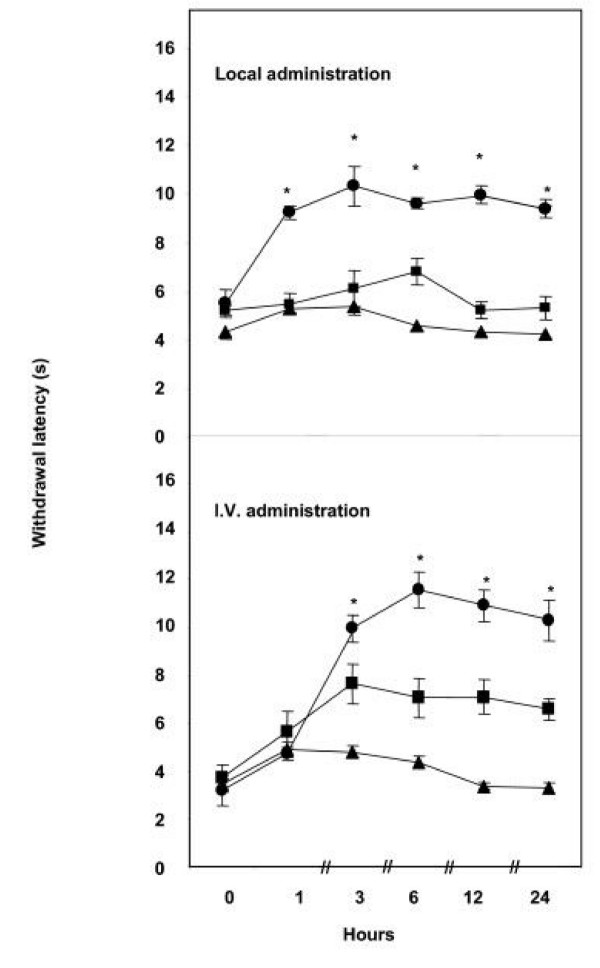
a) Withdrawal latencies measured 24 hours after intraplantar carrageenan injection (basal measurement = time 0) and after different hours from oATP (circle), or diclofenac (square), or indomethacin (triangle) oral administration (100 μM in 0.15 ml saline). Mean ± SEM of 7 rats. *p < 0.05 compared to diclofenac or indomethacin at the same time. b) Withdrawal latencies measured 24 hours after intraplantar carrageenan injection (basal measurement = time 0) and after different hours from oATP (circle), or diclofenac (square), or indomethacin (triangle) intravenous injection (100 μM in 0.15 ml saline). Mean ± SEM of 7 rats. *p < 0.05 compared to diclofenac or indomethacin at the same time.

In Fig. 3 the antihyperalgesic effect exerted by oATP (orally or intravenously injected) is compared to the effect exerted by diclofenac or indomethacin used at the same dose. Data show that the antihyperalgesic activity of oATP is significantly more elevated than that of diclofenac and indomethacin.

### Histologic expression of P2X7 receptors and HIS36

**Figure 4:**
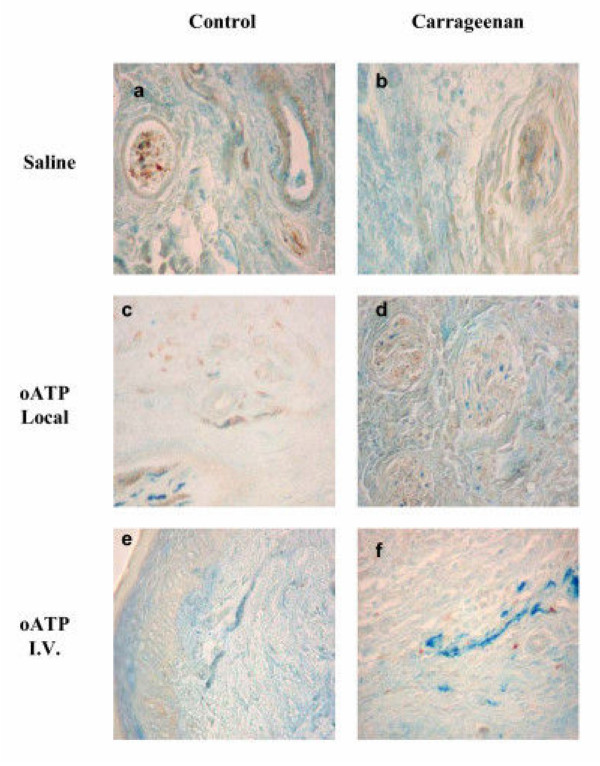
Immunohistochemistry of P2X7 receptors. Hind paw sections from control rats treated with saline (a) or oATP locally (c) or intravenously (e). After carrageenan induced inflammation, hind paws were submitted, to either saline (b), oATP local (d) or oATP intravenous (f) treatment. Notice the strong labelling on nerves and vessels in specimens treated only with saline, with variable presence of P2X7 which shows a general reduction after oATP local treatment, more evident after intravenous oATP treatment (original magnification 200x, P2X7 evidenced in brown, 3,3 diaminobenzidine, PGP 9.5 evidenced in blue, nithroblue tetrazolium). Data are representative of 5 experiments.

Each data is representative of 5 different performed experiments for each treatment. As reported in Fig. 4, an intense expression for P2X7 receptors was observed in nerve endings and peripheral nerves of control saline-treated rat hind paws. A similar result, but seldom and focally less intense, was observed in peripheral nerves in hind paws treated with carrageenan. In all specimens treated with carrageenan, an irregularly diffuse inflammatory infiltrate was present, with a prevalence of granulocytes and a variable amount of lympho-histiocytic cells displaying a weak and focal P2X7 positivity.

Hind paws treated with intravenous oATP, alone or after inflammatory reaction induced by carrageenan, presented a reduced expression of P2X7, either in nerves, or close to epidermidis, in dermis or intramuscolar. Few arterial vessels had a weak P2X7 positivity with a more prominent reduction in P2X7 expression in areas close to the inflammation site. Focally groups of cells with lympho-histiocytic cell morphology showed a strong cytoplasmatic granular positivity for P2X7.

Treatment with local oATP, alone or after inflammatory reaction induced by carrageenan, showed a focal, irregular and mild reduction of P2X7 expression, often more prominent in areas with phlogosis both in nerves and in vessels. In all specimens sweat glands revealed a faint but fairly diffuse P2X7 positivity.

**Figure 5:**
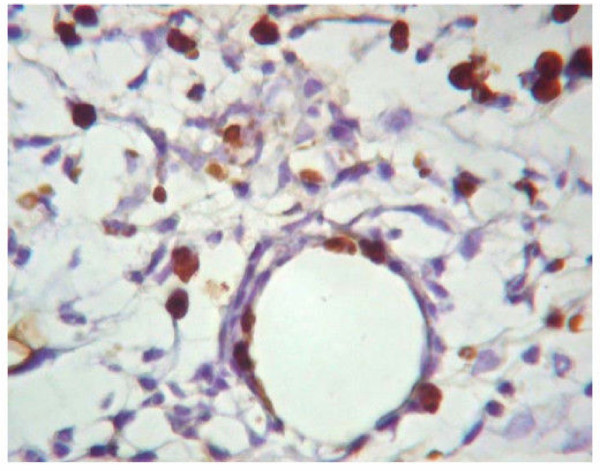
Presence of macrophages in rat inflamed paw section. In dermis and subcutis of carrageenan-treated hind paw a discrete, focal macrophagic (HIS36positive cells) infiltration is present (original magnification 250x; HIS36 evidenced with Dab). Dataare representative of 5 experiments.

Medium size isolated round cells with strong positivity for HIS36 and identified as macrophages were often present in a perivascular pattern in the dermis and subcutis of hind paws. They were more frequent in tissues with carrageenan treatment only (as reported in Fig. 5), few in rat hind paws with oATP local or intravenous administration and very few in those with saline only (data not shown). No differences in intensity or cellular immunostaining distribution were observed among the experiments. The summary of the immunohistochemical results are reported in table 1.

### Chemokine immunofluorescence expression

**Figure 6:**
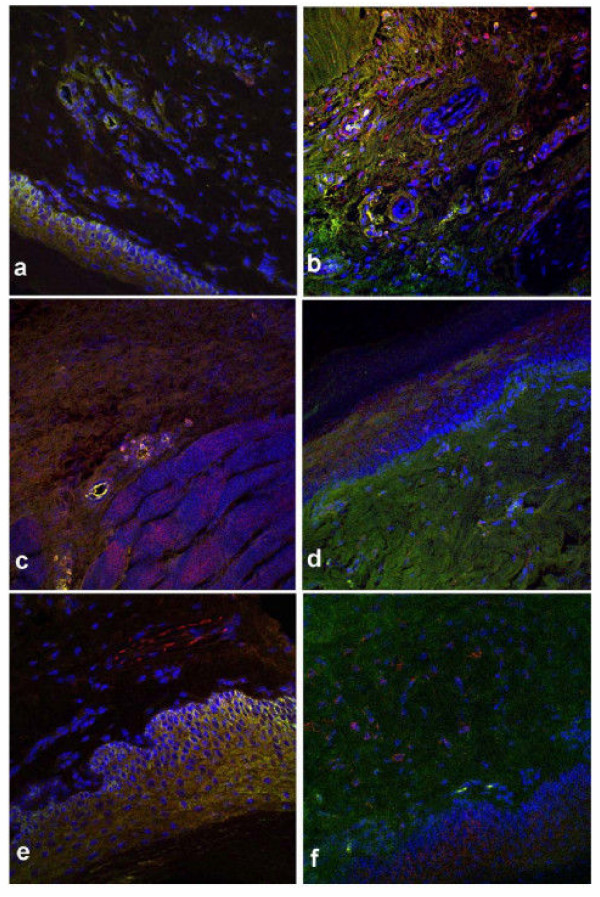
Confocal microscopy on rat paws. The sections are doubly labeled with anti- IP-10 and IL-8 mAbs revealed by FITC- (green) or TRITC- (red) conjugated secondary Abs respectively. Merged Free Projection Max of images seriesshows cytokine expression after saline (a), carrageenan (b), carrageenan+oATP local treatment (c), carrageenan+oATP intravenous treatment (d), local oATP treatment (e), intravenous oATPtreatment (f). Nuclei are stained with DAPI (blue) (original magnification 400x). Notice thepresence of activated cells in the derma in b and f, the endoluminal signal in dermal small vessels(a, c, e) and the absence of cytokine labeling in d (original magnification 400x).

Clinical findings about the development of a local inflammation in hind paws by carrageenan and the antiinflammatory effects of oATP were supported by immunofluorescence assessment of the presence of three inflammatory chemokines, commonly involved in inflammation-dependent immune response phenomena: MCP-1, IL-8 and IP-10. In all the processed rat paws, cytokine expression was confirmed in dermis and always absent in epidermidis. Any relevant expression of MCP-1 was not observed by confocal microscopy in the analyzed bioptic samples, excluding a weak label of arteriolar endothelium and isolated cells in reticular dermis in carrageenan inflamed specimen sections (data not shown). The control, submitted to saline only, failed to show fluorescent signals for all the three cytokines on both papillary and reticular layers of the dermis (Fig. 6a). Conversely, carrageenan-treated rat hind paw section samples expressed both IL-8 and IP-10 on dermis infiltrating cells, but not on vessel walls (Fig. 6b). A low expression of IP-10 on papillary dermis cells was observed on hind paws from rats treated with oATP alone, following either local (Fig. 6e) or intravenous injection (Fig. 6f). The IP-10 appeared co-localized with IL-8 on small vessel wall of dermis reticular layer in specimens from rat paws carrageenan-injected, then submitted to oATP local treatment (Fig. 6c). No significant chemokine labeling was assessed on specimens upon local treatment of rat paws with carrageenan and intravenous injection of oATP (Fig. 6d). The intravenous administration of oATP seemed more effective then the local injection in reducing the expression of chemokines in carrageenan-inflamed hind paws, according to clinical findings.

## Discussion

The hypothesis that ATP is a pain mediator [28] has supported the studies on its P2X receptors. Recently, ATP-mediated mechanical hyperalgesia has been shown to be decreased by the use of P2X3 receptor antagonists [29].

Based on our previous reports of the antinociceptive activity of oATP at the level of the hind paws in rats treated intraplantarly with CFA [3], we investigated the therapeutic effect of oATP in another model of rat paw inflammation, carrageenan-induced. The extracellular ATP released in inflamed tissues has pronociceptive activity [30,31]: it possibly acts by binding and activating the P2X receptors for ATP present on pain-sensing neurons. In addition, since P2X7 receptors are localized on many tissue components involved in inflammation, the binding and activation of such receptors by ATP could enable the same structures to release proinflammatory and pronociceptive mediators. Local treatment with oATP abrogates inflammatory pain possibly by inhibiting P2X7 receptors for ATP which are localized on nerve terminals and on vessels [2,3].

Paw inflammation is a multifactorial response due to proinflammatory chemokines which are responsible for the infiltration and activation of various leukocyte population in joint tissue. We analysed in parallel the expression of some chemokines (MCP-1, IP 10 and IL 8) and the expression of P2X7 receptors in the same inflamed tissues and their modifications due to oATP treatment. The analysis of the chemokine presence had the aim to evaluate wheter the use of oATP was able to influence the recruitment of circulating leukocytes. This feature was not shown by IL-lβ, a cytokine produced by activated macrophages, which fails to display chemoattractant properties, indicating only the state of macrophage activation. The three selected chemokines are described to be able to recruit the principal inflammatory cells: granulocytes (IL-8), monocytes/macrophages (MCP-1) and T lymphocytes (IP-10).

Our clinical results indicate that: 1) local treatment with oATP significantly relieves inflammatory pain in carrageenan-induced paw inflammation model, as in previously reported CFA model [3]; 2) oral and intravenous treatments with oATP are more efficient than local treatment in reducing the inflammatory pain; 3) intravenous treatment is more efficient than oral treatment; 4) the antinociceptive activity of a single dose of oATP lasts 24 hours; 5) the antinociceptive activity of oATP is more evident than that due to diclofenac or indomethacin in our experimental model. Based on the increased antinociceptive effect due to oATP intravenously administered with respect to that orally administered, we studied the expression of P2X7 receptors and of chemokines in inflamed tissues following local or intravenous oATP administration, e.g. in conditions of minimal or maximal antihyperalgesic oATP effect.

P2X7 receptors are involved in several processes concerning immunomodulation and inflammation. Previous reports show that the absence of P2X7 receptors alters cytokine production and leukocyte function and attenuates the inflammatory response [32,33]. In fact, high ATP concentrations were unable to promote IL-lβ extracellular accumulation from lypopolysaccharide (LPS)-activated blood samples derived from P2X7 receptor-deficient mice, in contrast with blood samples obtained from wild-type. In addition, P2X7 receptor-deficient mice were protected against clinical signs of mAb-induced arthritis [33]. Recent data indicate that, in response to ATP binding, the P2X7 receptors facilitate cation channel activation, non-specific pore formation, rapid changes in plasma membrane morphology (blebbing) and secretion of IL-1β from LPS-primed macrophages [34]. The "blebbing" induced by ATP was blocked by oATP. Blebbing was abrogated in the presence of Rho-effector kinase inhibitors, whereas ATP-induced IL-lβ release was unaffected, suggesting different signalling pathways for P2X7 receptors. In addition, the P2X7 receptor-protein complex comprises several distinct identified proteins, with different regulatory capacities [35], indicating a complexity for the P2X7 receptor functions.

We showed the antinociceptive activity of oATP, which is able to block the P2X7 receptor [1], in rats bearing acute paw inflammation. The question arises about the mechanisms by which the oATP-induced inhibition of P2X7 receptor activation are able to relieve inflammatory pain. Our previous data suggested the possibility that the block of P2X7 receptors expressed by nerve terminals could be useful to inhibit pain transmission [3]. In inflamed paws the expression of P2X7 receptors was less evident in oATP-treated than in untreated rats, independently on the route of oATP administration. Noteworthly, such expression was reduced at the level of nerve terminals and of vessels close to inflammation site, more than at the level of the immune cells (see Fig. 4 and 5). The expression of MCP-1 was weakly evident only in carrageenan-inflamed rat paws at the level of arteriolar endothelium and of some dermal cells; in the same structures we observed by immunohistochemistry an increased number of macrophages (HIS36 positive cells). MCP-1 was absent and only few macrophages were present in paws from oATP-treated rats.

These data confirm the strict correlation between P2X7 expression by leukocytes and relative inflammatory response and underline that the reduction of inflammatory effects due to oATP administration is similar to that observed in P2X7 receptor deficient mice [33]. Carrageenan-treated rat paws expressed IL-8 and IP-10 on dermal infiltrating cells, whereas a low expression of IP-10 on dermal cells followed oATP treatment alone (both local and intravenous). IP-10 and IL-8 were co-localized on the wall of small vessels in carrageenan-inflamed paws of intraplantarly oATP-treated rats. Moreover, no chemokine label was observed in carrageenan-inflamed paws of intravenously-oATP-treated rats. Such results indicate that oATP treatment significantly reduced chemokine expression at vascular rather than at infiltrating cell level. In fact oATP local treatment of carrageenan-inflamed paws completely abrogated MCP-1 expression, whereas the intravenous treatment with oATP abrogated the expression of all the three chemokines in inflamed paws. In particular, the abrogation of MCP-1 production by oATP could be related to the oATP-induced block of P2X7 function on activation of endothelial and immune cells, which can become ineffective to produce MCP-1.

In our results we underline the relation between the absence of recruited macrophages and the absence of MCP-1 expression in the inflamed paws of intravenously oATP-treated rats.

Other studies demonstrate the presence of P2X7 receptors on nervous and vascular structures. In fact, P2X7 immunoreactivity has been shown in rats at the level of glial cells of gastrointestinal musculature, in mienteric and submucosal ganglia (where perineuronal nerve endings appeared brightly labeled) [36]. In addition, pharmacological identification of P2X1, P2X4 and P2X7 nucleotide receptors has been evidenced in the smooth muscle of human umbilical cord and chorionic blood vessels [37]. Moreover, LPS-activated endothelial cells have been shown able to secrete ATP, via P2X7 receptors, and to release IL-lα [38].

During carrageenan-induced acute inflammation ATP is released by damaged cells. When ATP activates cells involved in the inflammatory process and bearing P2X7 receptors, other mediators and ATP molecules are released by these cells. Indeed, oATP could block such amplifying mechanism of inflammation and also downregulate P2X7 receptor expression, especially in vessels and nerves close to the site of inflammation (see Fig 4). This hypothesis is supported by the fact that chemokine expression is reduced by oATP treatment. We hypothesize that oATP, by blocking the P2X7 receptors present on nerves and endothelial cells, could regulate some effects of ATP (having pronociceptive functions when released) on these structures. The effect of oATP was greater when given orally or intravenously instead of locally, possibly because the oral and intravenous administrations of oATP could permit its systemic diffusion. Thus oATP could reach sites distal to the site of inflammation and so enhance the contact with circulating leukocytes bearing P2X7 receptors. Such contact favours the inactivation of immune cells, which are able to produce inflammatory pronociceptive mediators. In addition, the most antinociceptive activity obtained when oATP was intravenously injected (instead of locally or orally) could indicate that the molecule crosses the blood-brain barrier and that it may also have central effects and not only peripheral effects. It is also possible that oATP blocks other receptors than P2X7, e.g. P2X1 and P2X2 [4]. Effectively, human P2X1 and P2X7 receptors are co-expressed in several cell types such as lymphocytes or epithelial cells [39], and oATP was found to be ineffective in separating P2X1 receptor current from the P2X7 current. In addition, none oATP activity on P2X4 has been reported. However, the involvement of P2X7 is also assured by the fact that P2X3-/- mice did not display a difference in thermal hyperalgesia to wild type controls in the carrageenan model of inflammation [40]. Our data suggest that the potential analgesic and also the anti-inflammatory properties of oATP might have significant therapeutic potential in reducing inflammatory pain. In addition, the more efficient antinociceptive activity of oATP with respect to that of some known NSAIDs is helpful, in consideration of NSAID direct cytotoxic effects [41].

Recently, it has been demonstrated that oATP can reduce pro-inflammatory TNFα-induced signalling in HEK293 cells, that lack of P2X7 expression. Such and other reported results possibly indicate that oATP interfere with the activation of signalling pathways involved in the inflammatory response, independently on oATP action on P2X receptors [42], thus showing a mechanism independent of the expression or activation of known P2 receptor subtypes. However, the involvement of the P2X7 receptors in the inflammatory pain has been suggested by other results [43]. In fact, mice lacking P2X7 receptors did not display inflammatory and neuropathic hypersensitivity to both mechanical and thermal stimuli and were impaired in the ability to release IL-1 beta, IL-10 and MCP-1, the latter in agreement with our findings, obtained with the administration of oATP in rats bearing paw inflammation. All data suggest that P2X7 receptors exert a role in both acute and chronic inflammations.

## Conclusion

Our results show that oATP systemically administered removes hyperalgesia induced by carrageenan in rat paws. Tha data suggest the possible use of oATP to reduce the inflammatory pain.

## Methods

### Animals

The procedures followed the guidelines of the International association for the Study of Pain [24]. Male Wistar rats from Harlan Italy (Corezzana, Milano, Italy) weighing about 250 g were used. They were housed in groups of three per cage, allowed free access to water and food and exposed to a 12/12-hour light/dark cycle, and acclimatised to the laboratory at least 5 days prior to the experiments.

### Induction of inflammation

Following brief exposure to halothane (5%) anesthesia (Hoechst, Milano, Italy), inflammation was induced by the injection of 0.1 ml 1% carrageenan (λ-carrageenan, Sigma-Aldrich, Milan, Italy) in sterile saline in the right hind paw of the rats. Maximal incidence of inflammation (e.g. the hind paw edema) occurred 3 hours after carrageenan injection, as previously reported [25].

### Administration of oATP

Three hours following carrageenan injection, the rats were treated with oATP (Sigma-Aldrich) dissolved in 0.15 ml sterile saline, whereas control rats received the drug vehichle alone (0.15 ml sterile saline). Firstly, 3 different doses oATP (50 μM, 100 μM, 200 μM, in 0.15 ml, respectively) were administered: 1) locally, by intraplantar injection in the right hind paw; 2) orally, by using an orogastric catheter; 3) intravenously, through the left femoral vein. The injections were performed under brief halothane anestesia and the antinociceptive effect of oATP was measured 3 hours following its administration. Since a more significant antihyperalgesic effect was observed by using 100 μM oATP (see Results section), such dose was selected among the tested doses to perform further experiments. To perform histologic evaluations (by immunohistochemistry or by confocal analysis), oATP was administered locally or intravenously; in fact, as shown in the Results section, these routes of administration respectively showed minimal or maximal antihyperalgesic effect (as clinically evidenced).

### Reduced thermal hyperalgesia measure

The observers of the thermal hyperalgesia measurements were blinded. The method of Hargreaves was used to assess the hind paw nociceptive thresholds to thermal stimuli [26]. We used a plantar test apparatus (Ugo Basile, Comerio, Italy). Briefly, the rats were placed in a clear plastic chamber and left to acclimatize for 5 minutes before testing. Radiant heat stimulus was induced by light from a 8 V-50 W halogen bulb (64607 OSRAM), delivered to the plantar surface of the rat's hind paw through the base of the plastic box; the beam was about 12 mm in diameter. The rats were familiarized with the handling procedure 3 days before the antinociceptive test. Time taken by the animal to withdraw its right hind paw was measured before and after oATP injection. Reduced thermal hyperalgesia was defined as a significant increase in the withdrawal latency compared to the basal measurement. The values of thermal hyperalgesia were determined at 1, 3, 6, 12 and 24 hours after oATP injection. The same procedure was used to test the antihyperalgesic effect of diclofenac and indomethacin orally or intravenously administered at the same dose of oATP (e.g. l00 μM in 0.15 ml saline)

### Confocal analysis

Rat right hind paws were sampled and processed for cryostatic inclusions. For any treatment, 5 different experiments were performed. The treatments included: saline, carrageenan, oATP alone, and carrageenan ± local or intravenous oATP administration. The same hind paws were also used for immunohistochemistry.

Biopsies were fixed in 4% paraformaldehyde in Dulbecco's PBS (DPBS) then cryoprotected in 20% sucrose in DPBS, embedded in Tissue Tek medium and snap-frozen by immersion in liquid nitrogen. Pseudo-seriated l0 μm thick sections were submitted to indirect immunofluorescence staining by using mouse monoclonal antibodies (mAbs) against mouse α MCP-1 (B-B4 RPE, Groningen, The Netherlands)ÙIP-10 (Peprotech EC Ltd., London, UK), and IL-8 (Pharmingen, San Diego, CA). The sections were incubated in blocking solution (1% BSA in DPBS, 30 minutes, RT), then in primary Ab diluted in blocking solution (2 hours, RT), finally, after 30 minutes of DPBS washing, in conjugated secondary Ab (Rabbit-anti-Mouse Ig-FITC conjugated, DAKO, Milano, Italy) in DPBS (30 minutes, RT). Double staining was realized by superposing the same procedure with a different primary Ab and a TRITC-labelled secondary Ab. The 3 possible couplings of primary Abs were tested and single staining served as controls. DAPI nuclear staining (0.2 nM, 20 minutes, RT) followed the immunostaining steps.

The sections, mounted with Fluorsave (Calbiochem, Merck Eurolab Srl, Milano, Italy) were analysed with the support of a confocal microscope Leica TCS SP2 (Leica Microsystems, Heidelberg, GmbH); 3D maximum projections were obtained from single channel-collected series of images, subsequently they were superposed by Adobe Photoshop software.

### Immunohistochemistry

Materials and methods for immunostaining have been previously described [3]. Immunostain was perfomed on all specimens by using two different procedures. Sections (4 μm-thick) were prepared on slides pretreated with poly-L-lysine (Sigma Diagnostic Inc., St Louis, USA). A double staining method was applied to examine the relationship between P2X7 and cutaneous nerve fibers using anti-P2X7 receptor purified polyclonal antibody (Chemicon International, Inc., Temecula, CA) and the monoclonal antibody Protein Gene Products 9.5 (Novocastra Laboratories Ltd, Newcastle, UK). The anti-P2X7 polyclonal antibody was directed against a c-term antibody (epitope 567–595), which has been previously shown highly selective [27]. Binding was revealed by 3',3'-diaminobenzidine (Liquid Dab, BioGenex, San Ramon, CA) for P2X7 receptor and by 5-bromochloroindoxyl phosphate and nitro blue tetrazolium chloride (DAKO, Copenhagen, Denmark) for PGP 9.5. The two procedures were performed alternatively, on both antibodies. Sections were counterstained with Green Light. To obtain a negative control, the primary antibodies were routinely omitted.

In a separate setting of immunostainings we used a mouse monoclonal antibody HIS36 (Santa Cruz, California, USA) specific for rat macrophage subset. All the immunostained slides were viewed in a blinded fashion.

### Statistical analysis

The withdrawal latency data (expressed as mean ± SEM) were analyzed using a two-way analysis of variance followed by Dunnett's test. Significance was assumed when P < 0.05.

## Abbreviations

oATP, periodate oxidized ATP; MCP-1, monocyte chemoattractant protein-1; VEGF, vascular endothelial growth factor, IP-10, interferon gamma-inducible protein-10; IL8 interleukin 8

## Authors' contributions

AF carried out clinical experiments and statistical analysis, GdA and EDC carried out the immunohisochemical studies, CF and PT carried out the confocal analysis, JSF participated in the sequence alignment and MEF performed the design of the study and has written the manuscript.

## Figures and Tables

**Table 1:** Immunohistochemical results in rat hind paws after induction of inflammation by carrageenan

Treatment	P2X7 receptor nerve expression	P2X7 receptor vascular expression	P2X7 receptor expression in lympho-histiocytic cells	Quantity of macrophages (HIS36+)
Saline only	*Strong*	*Strong*	*Weak*	*Very few*
Carrageenan only	*Strong, locally moderate*	*Strong, focally moderate*	*Weak*	*Focally in discrete amount*
Carrageenan + OATP intraplantar injection	*Moderate to weak*	*Moderate to weak*	*Weak to moderate*	*Few*
Carrageenan + oATP intravenous administration	*Weak to absent*	*Very weak (absent in area close to inflammatory infiltrate)*	*Moderate, focally strong*	*Few*

Note. A discrete amount of granulocytes are present in carrageenan-treated hind paws.
